# Metformin extends the chronological lifespan of fission yeast by altering energy metabolism and stress resistance capacity

**DOI:** 10.1093/femsyr/foad018

**Published:** 2023-03-20

**Authors:** Ceren Şeylan, Çağatay Tarhan

**Affiliations:** Department of Molecular Biology and Genetics, Faculty of Science, Istanbul University, 34134 Vezneciler, Istanbul, Turkey; Department of Molecular Biology and Genetics, Faculty of Science, Istanbul University, 34134 Vezneciler, Istanbul, Turkey

**Keywords:** fission yeast, metformin, chronological lifespan, aging

## Abstract

The antiaging properties of metformin used for the treatment of type-2 diabetes mellitus have been studied extensively, but there is more to discover regarding underlying mechanisms. Here, we show that metformin significantly prolongs the chronological lifespan (CLS) of *Schizosaccharomyces pombe* through mechanisms similar to those observed in mammalian cells and other model organisms. While the presence of metformin in the medium caused an increase in carbohydrate consumption and ATP production, it reduced reactive oxygen species production and alleviate oxidative damage parameters such as lipid peroxidation and carbonylated proteins. We also tested whether the effect of metformin changed with the time it was added to the medium and observed that the lifespan-prolonging effect of metformin was related to the glucose concentration in the medium and did not prolong lifespan when added after glucose was completely depleted in the medium. On the other hand, cells inoculated in glucose-free medium containing metformin also showed extended lifespan suggesting that mechanisms other than that solely depend on glucose availability may be involved in extending the lifespan. These results suggest that metformin prolongs lifespan especially affecting energy metabolism and stress resistance capacity and that fission yeast can be effectively used when investigating the antiaging mechanisms of metformin.

## Teaser

Metformin increases the lifespan of fission yeast, as seen in various organisms, by increasing glucose consumption and energy production, reducing oxidative damage and providing resistance to stress, and it also shows this effect in glucose-free medium.

## Introduction

Aging is defined as a deterioration process that causes loss or impairment of physiological integrity and function at the level of molecules, cells, organs, and organisms, and as a result, it increases the vulnerability to death (López-Otín et al. 2013). Because many systems at the molecular, cellular, and organismal levels are affected synchronously, establishing a cause–effect relationship is not easy. Although today we assume that the phenomenon of aging is suitable for scientific research, only 30 years ago it was hotly debated (Zainabadi 2018). Studies on aging have experienced unprecedented development in recent years, especially with the discovery of controlling the aging process, albeit to a certain extent, by evolutionarily conserved genetic and biochemical mechanisms. These studies have yielded that it is important to consider the multipotent factors that will affect many molecular and cellular processes in slowing down aging, where a single initiating mechanism and period cannot be determined precisely.

Metformin, which is one of these factors, has become the most prescribed drug in the world as a glucose-lowering agent after it was approved by the FDA in 1994 for use in type-2 diabetes. The antihyperglycemic role of metformin is associated with its effect on glucose metabolism as a suppressor of hepatic gluconeogenesis, especially by inhibiting mitochondrial glycerophosphate dehydrogenase and changing hepatocellular redox state, resulting in a reduction of glucose formation from lactate and glycerol (Madiraju et al. 2014, Bailey 2017). On the other hand, it has been shown that the reduction in hepatic glucose production by metformin is a result of AMP-induced inhibition of fructose-1,6-biphosphatase-1, an enzyme that controls the rate of gluconeogenesis (Hunter et al. 2018). Although all its molecular mechanisms are not yet fully elucidated, its ability to promote phosphorylation and AMPK activation is thought to be central to metformin’s mechanism of action, resulting in the inhibition of gluconeogenic genes (An and He 2016). Activation of AMPK, which is considered an important regulator of cellular metabolism, affects many pathways targeting lipid metabolism, mitochondrial biogenesis, autophagy, cell growth, and circadian rhythm as well as glucose metabolism (Hardie 2015). Due to these multifaceted effects, its potential in processes other than diabetes such as cancer, immunoregulation, and aging has been extensively studied for a long time (Pearce et al. 2009, Martin-Montalvo et al. 2013, Sui et al. 2015).

Studies on different model organisms and human cell lines have illuminated the role of metformin in targeting aging mechanisms. Metformin reduces insulin levels and IGF-1 signaling (Liu et al. 2011), and activates AMPK signaling (Foretz et al. 2010, Zheng et al. 2012, Lien et al. 2014, Cho et al. 2015, Duca et al. 2015, Lu et al. 2015), inhibits mTOR pathway (Kickstein et al. 2010, Nair et al. 2014, Pérez-Revuelta et al. 2014), inhibits mitochondrial complex 1 in the electron transport chain and causes reduction of endogenous reactive oxygen species (ROS) production (Batandier et al. 2006, Zheng et al. 2012, Bridges et al. 2014), and DNA damage (Algire et al. 2012). Metformin also positively affects metabolic and cellular processes closely related to aging, such as inflammation (Saisho 2015), autophagy (Xie et al. 2011, Song et al. 2015), and cellular senescence (Jadhav et al. 2013). However, it is not certain whether this multifaceted effect of metformin in the cell is associated with triggering a single mechanism related to aging and sequential cellular events, or whether it occurs because it can affect many targets in different pathways at the same time (Barzilai et al. 2016). Therefore, it is important to elucidate that through which genes and cellular pathways metformin influence the aging process.

On the other hand, metformin did not show similar effects on lifespan and healthspan in all studies with different model organisms. The beneficial effects of metformin on nematode lifespan do not appear to be evolutionarily conserved in fruit flies (Novelle et al. 2016). For example, metformin treatment to *Drosophila melanogaster* caused strong activation of AMPK and decreased lipid stores, but did not affect longevity (Slack et al. 2012). Studies showing that metformin prolongs lifespan have mostly examined animals given metformin when they are young. Few studies in older animals have either failed to detect metformin-induced increases in lifespan (Anisimov et al. 2011, Alfaras et al. 2017) or demonstrated toxicity induced by high-dose metformin (Martin-Montalvo et al. 2013, Thangthaeng et al. 2017). It has been observed that 50 mM metformin, which prolongs the lifespan of young nematodes, causes moderate toxicity in middle-aged nematodes (Onken and Driscoll 2010). The dose and duration of administration of metformin, therefore, require serious consideration of several criteria.

With the increasing use of yeasts in aging studies, it has been proven that the pathways affecting lifespan in various eukaryotes, including mammals, are also conserved in yeasts (Kaeberlein et al. 2007). For example, calorie restriction, which is the most used experimental intervention to increase lifespan in numerous model organisms, also prolongs both replicative (RLS) and chronological lifespan (CLS) of *Saccharomyces cerevisiae* (Fabrizio et al. 2005, Kaeberlein and Kennedy 2005). CLS has also been extensively investigated in *Schizosaccharomyces pombe*, which has become a popular model organism in aging studies in recent years. These studies suggest that CLS could be associated with cellular processes such as nutrient signaling (Roux et al. 2006, Ohtsuka et al. 2008, Chen and Runge 2009), mitochondrial activity (Zuin et al. 2008, Roux et al. 2010, Stephan et al. 2013), ROS production, stress resistance (Mutoh and Kitajima 2007, Zuin et al. 2010), proteasome activity, and autophagy (Takeda et al. 2010).

On the other hand, only a few studies have been done with metformin in yeasts, while to the best of our knowledge, there is no such study has been done in *S. pombe* (Borklu-Yucel et al. 2015, Avelar-Rivas et al. 2020). Here, we aimed to determine the effects of metformin on the lifespan and some cellular parameters such as carbohydrate consumption, ATP production, intracellular oxidation, and stress resistance in *S. pombe*. A total of 25 mM metformin causes an increase in the lifespan of *S. pombe* by ∼50%, but the time of addition of metformin to the medium resulted in different effects. While metformin significantly increased carbohydrate consumption and stress resistance, its effects on ATP production, intracellular ROS, and carbonylation depended on the day of measurement. These results may shed more light on the antiaging mechanisms of metformin, prove that *S. pombe* is a good candidate model organism for metformin studies, and contribute to the aging studies in *S. pombe*.

## Materials and methods

### Organism and media

In all experiments, *S. pombe* wild-type strain 972^−^ from a single colony grown on a yeast extract agar (YEA) plate was used. Because synthetic dextrose (SD) medium was shown to be an appropriate condition for lifespan experiments and cells grown in SD with excess glucose showed the evolutionarily conserved response to lifespan (Chen and Runge 2009). For this reason, SD medium with 3% glucose was used for lifespan analysis and other experimental investigations. 1,1-dimethyl biguanide hydrochloride, %97 (Sigma) was used for metformin treatment. For all experiments, the initial cell density was 5 × 10^4^ cells/ml and cells were inoculated in 25 ml of SD medium in a 125 ml flask, and they were grown at 30°C, in an orbital shaker at 180 rpm. For lifespan experiments, starting from day 0, aliquots of cultures were taken every 4 days, serially diluted in sterile distilled water, streaked onto YEA plates, and grown at 30°C for 4 days, then colonies were counted. Each experiment was done at least twice. For other experiments, indicated amounts of samples were taken as needed for each experiment from the second and third days of inoculation. In all experiments, duplicate assays were performed and error bars show the ranges of the values. Unless otherwise stated, metformin was added to the media from the beginning in all experiments.

### Determining carbohydrate consumption

Carbohydrate consumption in metformin-treated cells was examined using the Anthrone method (Kamlage 1996). Briefly, 1 ml of suspended cell cultures were filter-sterilized and transferred to glass tubes. A volume of 2 ml of 75% H_2_SO_4_ solution was added into the filtrates and vortexed, then the tubes were immediately placed on ice to prevent them from overheating. A volume of 4 ml of anthrone solution was added to each of the mixtures and vortexed. The glass tubes were left in boiling water for 15 min. Then the tubes were cooled down at room temperature and the spectrophotometric measurement was made at 540 nm.

### Measurement of ATP production

Intracellular ATP concentration was measured using ATP colorimetric assay kit (Sigma-Aldrich/MAK190). A total of 10^6^ cells were collected and centrifuged, washed twice with sterile distilled water, and disrupted in 100 μl ATP assay buffer using a dismembrator. Measurements were performed according to manufacturer instructions.

### Measurements of intracellular total oxidation and lipid peroxidation levels

The levels of ROS formed in the cells were determined with a fluorescent dye, 2',7'-dichlorofluorocein (DCFH) (Sigma-Aldrich). A volume of 1 ml of suspended cell cultures were centrifuged for 5 min at 5000 rpm. After the supernatant was removed, 1 ml of 3% Yeast Extract Liquid (YEL) medium was added to the pellet and the pellet was resuspended. The solution was transferred to 24-well plates. A volume of 4 μl DCFH was added to the solutions in a dark environment with a final concentration of 50 μM, and the plate was wrapped with aluminum foil and incubated for 1 hour in a shaking incubator at 30°C. At the end of incubation, cells were kept on ice for 5 min. After centrifugation and washing two times, cells were incubated in 400 μl of phosphate buffered saline (PBS) (pH:7.2). A volume of 200 μl of samples were taken and transferred into a black fluorescent plate and kinetic readings for 2.5 hours were made with BioTek® Instruments, Inc. FLx800 Spectrofluorometer Microplate Reader device and KCjuniorTM program. Results were expressed as the relative fluorescent intensity/10^3^ cells.

The levels of intracellular thiobarbituric acid reactive substances (TBARS) were measured for the detection of lipid peroxidation (Aust 1994). Accordingly, 1 ml of thiobarbituric acid reagent [0.25 M HCl, 15% (w/v) trichloroacetic acid, and 0.375% (w/v) thiobarbituric acid] was added to 500 μl of samples taken from suspended cell cultures, incubated in a boiling water bath for 15 min. After cooling down at room temperature cell debris was centrifuged. The absorbance of the supernatant was measured at 535 nm against a blank solution containing 0.5 ml of distilled water. TBARS content was calculated from a standard graph prepared with MDA bis-dimethyl acetal and the results were expressed as µg MDA/10^3^ cells.

### Spectrophotometric detection of protein carbonyls

The carbonyl content of the samples was determined according to the method described by Levine et al. (1990). First, 25 ml of cultures were centrifuged, washed twice with sterile distilled water, and resuspended in 0.5 ml of radioimmunoprecipitation (RIPA) buffer. After the addition of 1 M phenylmethanesulfonyl fluoride (PMSF), cells were lysed using a dismembrator with the aid of 0.5 mm glass beads. Protein concentrations were determined using SMART^TM^ BCA protein assay kit. Protein samples were treated with 0.1 ml of 2,4-dinitrophenylhydrazine (10 mM in 2 M HCI, Sigma-Aldrich) and incubated in the dark at room temperature for 1 hour. Proteins were precipitated with 0.1 ml of 20% trichloroacetic acid followed by a washing step with 0.2 ml of ethanol–ethyl acetate (1:1) and dissolving in 1 ml of solubilization buffer (6 M guanidine, 20 mM potassium phosphate, pH 2.3). Samples were incubated in the dark at 37°C for 1 h and centrifuged at 5000 rpm for 5 min. Spectrophotometric measurements for each sample were done at 380 nm.

### Assessment of the protective role of metformin against hydrogen peroxide and heat stress

To evaluate the resistance of cells grown in metformin supplemented and unsupplemented media to H_2_O_2_ and heat stress, we treated 1 ml of these cells with 100 and 300 mM H_2_O_2_ for 90 min and 55°C for 5 and 25 min (Chen and Runge 2009). For the oxidative stress assessment,1 ml of samples (∼10^7^ cells) were centrifuged at 5000 rpm for 5 min and the cells were then resuspended in 0.5 ml of H_2_O_2_ and incubated in an orbital shaker at 30°C for 90 min. Cells were washed once with the same amount of sterile distilled water and resuspended in 0.5 ml of sterile Milli-Q water. Cells were serially diluted from 1- to 10^4^-fold and 5 µl of each dilution was spotted on YEA plates and incubated at 30°C for 4 days.

Heat stress was applied to the cells that were suspended in preheated sterile Milli-Q water. For this, microcentrifuge tubes containing cells were incubated in a water bath at 55°C for 5 or 25 min. Then the tubes were placed on ice for 2 min and cells were spotted on plates as mentioned before.

## Results

### Effects of metformin on the CLS of *S. pombe*

**Figure 1.**
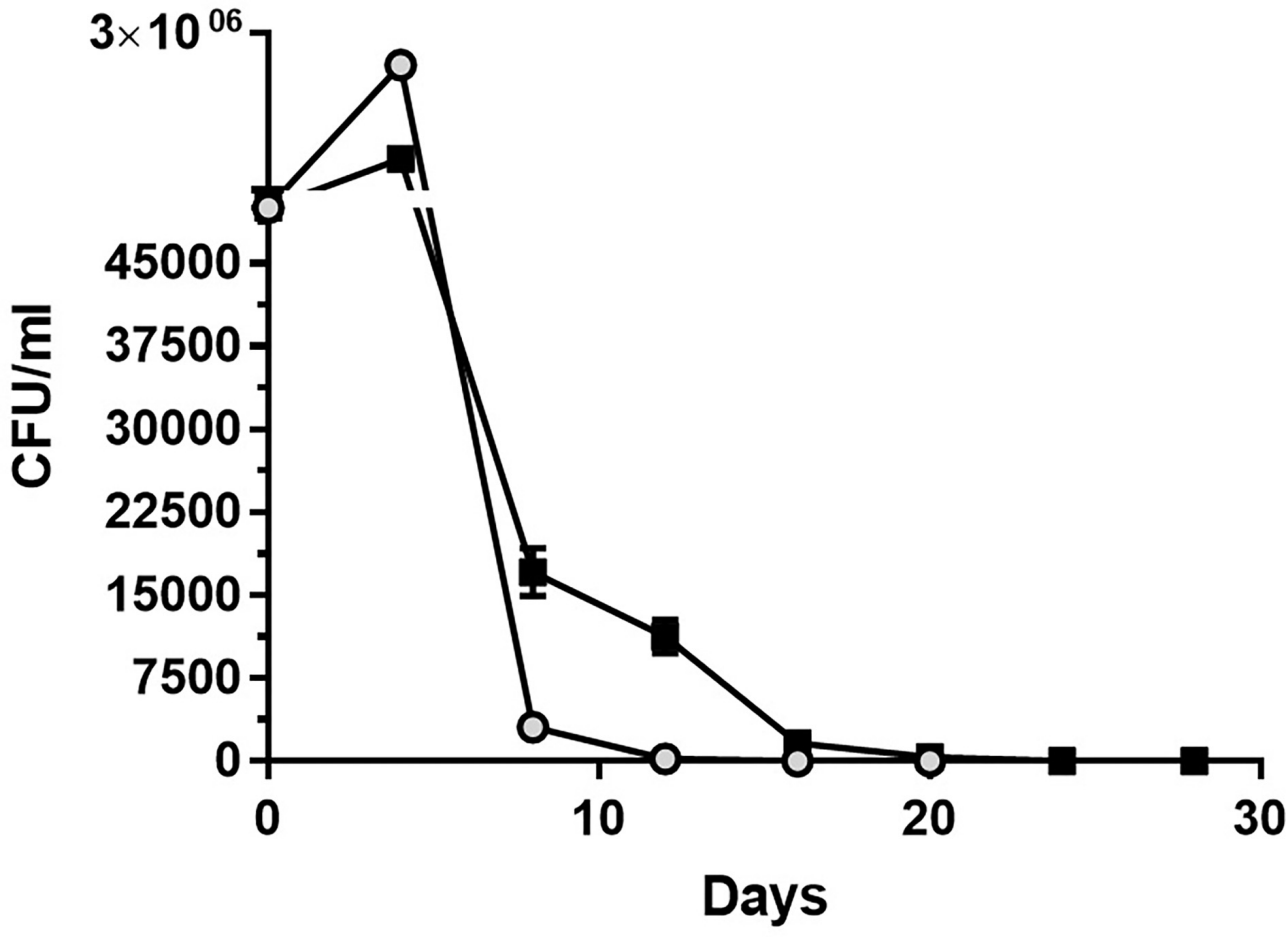
Metformin treatment lengthens the CLS of *S. pombe* grown in the 3% SD medium. Cells were seeded at 5 × 10^4^ cells/ml in a 25-ml culture and grown at 30°C. Samples were taken every 4 days and plated on the rich medium and colony-forming units or CFU per ml were assayed. The line with black squares represents metformin-treated cells, while the gray-circled graph represents untreated cells. Duplicate assays were performed and error bars show the ranges of the values (some error bars are too small and can not be visible on this scale).

First, to determine the effect of metformin on the chronological aging of *S. pombe*, a sample was taken every 4 days from SD medium with 3% glucose-containing 0 mM (control group) and 25 mM metformin and spread onto solid rich media, and colony counting was performed. It was observed that the lifespan of metformin-treated cells was ∼50% longer than the cells in the control group (Fig. 1)

**Figure 2.**
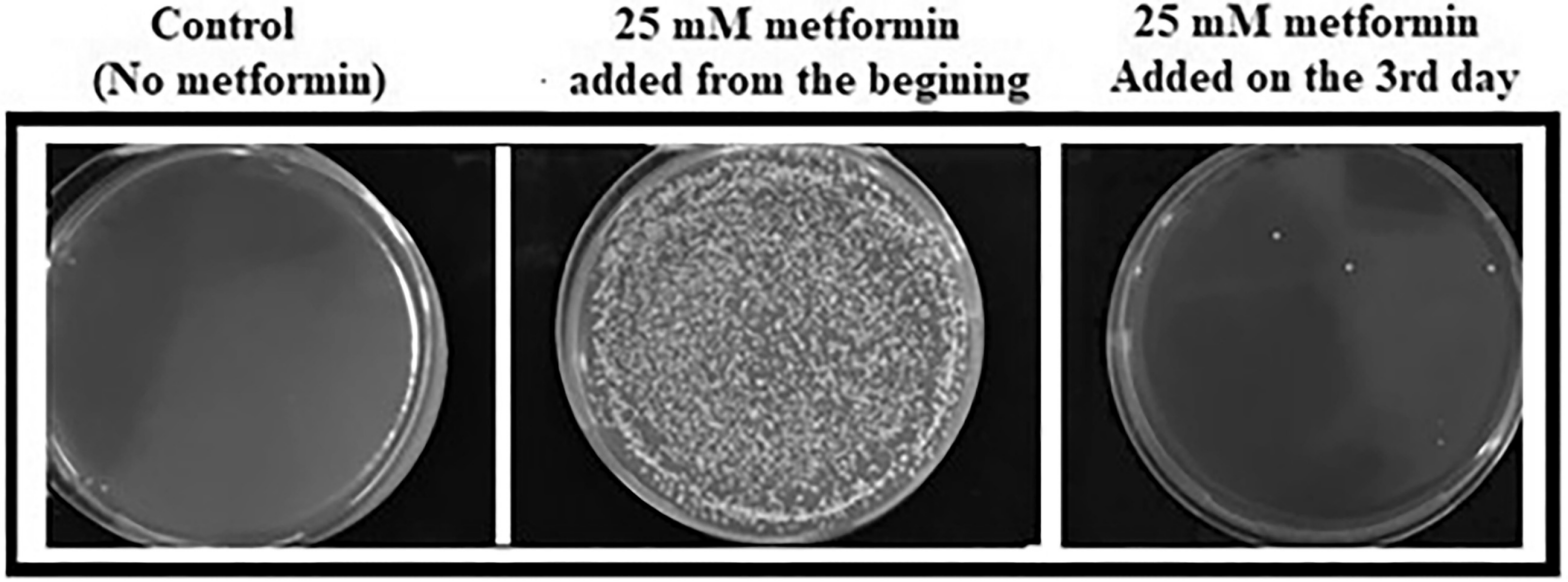
Metformin addition to the 3% SD medium on the third day of the culture did not prolong the lifespan (on the right). The lifespan of these cells was similar to the cells in the control group (on the left). As expected, adding metformin from the beginning (middle), caused a colony number profile (∼10 000 cells), i.e. compatible with the relevant graph in Fig. 1. Results were obtained by spreading directly 100 µl of samples from three different conditions onto a rich medium on the 14th day.

The effect of adding metformin to the medium on different days on *S. pombe* cells was also investigated. For this purpose, metformin was added to the medium at the start of the culture, first, second, and the third day of inoculation. Adding metformin on the third day did not increase the lifespan, and the lifespan of these cells was similar to untreated cells (Fig. 2).

**Figure 3.**
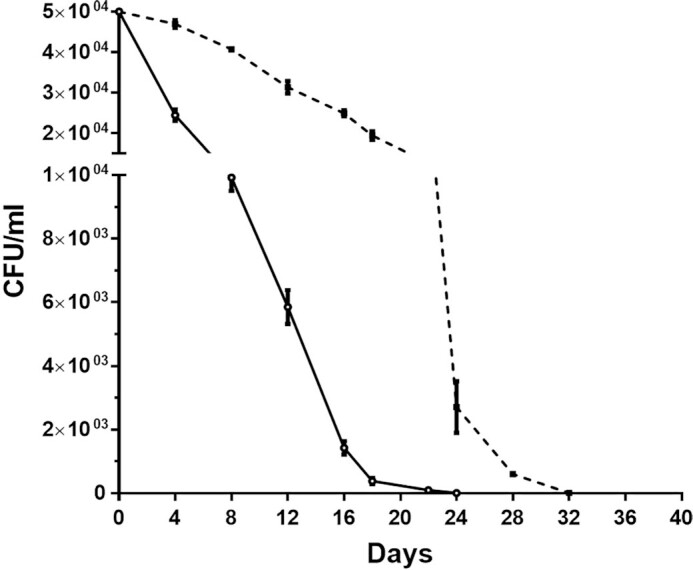
Metformin increased the lifespan of cells grown in the glucose-free medium. Except for the medium, the monitoring method was the same as the CLS assay above. The dotted line with black squares on the graph represents metformin-treated cells, while the solid line with gray circles represents untreated cells. Duplicate assays were performed and error bars show the ranges of the values (some error bars are too small and can not be visible on this scale).

Based on this finding, we reasoned that the lifespan-prolonging effect of metformin might be related to glucose availability in the medium. From this point of view, we cultivated cells in a glucose-free SD medium and treated them with metformin to understand whether metformin had the same effect in this condition. It is known that cells in media where there is no carbon source shift to the quiescent state. Understanding whether metformin will increase lifespan when cells are in this condition may provide a clue that metformin protection occurs through other mechanisms. Cells grown on glucose-free media had a longer lifespan than cells grown on media containing 3% glucose. On the other hand, we observed that when metformin was applied to these cells, the lifespan was even longer (∼33%, Fig. 3). These data suggest that under these conditions, metformin might lead to longevity by other mechanisms that do not necessarily depend on glucose availability.

### Effect of metformin on carbohydrate (glucose) consumption

**Figure 4.**
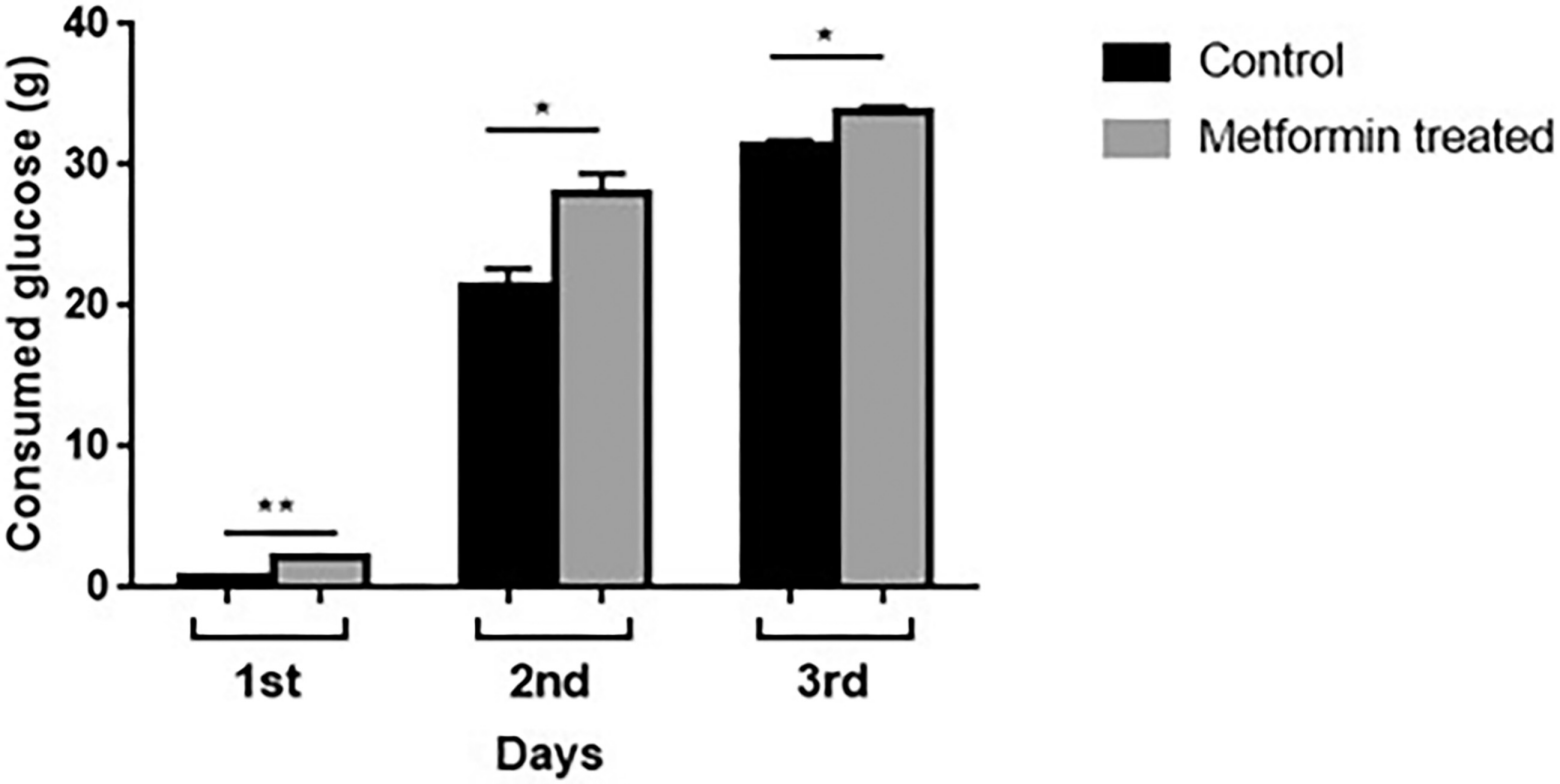
Metformin increased glucose consumption in cells grown in the 3% SD medium. A volume of 1 ml of cultures were taken once every 24 hours for 3 days and transferred to glass tubes. After adding and mixing 2 ml of chilled 75% H_2_SO_4_, 4 ml of anthrone solution was added and the mixture was vortexed and then boiled at 100°C for 15 min. After the tubes cooled down to room temperature the optical densities were measured at a wavelength of 540 nm. Assays were performed on three separate cultures for each condition. *P < .05, **P < .01.

To understand the effect of metformin on glucose utilization of *S. pombe* cells, glucose consumption in the control and metformin groups was measured and the amount of carbohydrate remaining in the medium was calculated. Accordingly, 25 mM metformin increased glucose consumption. At the end of the third day, the control group used ∼92% of the glucose in the medium, while the metformin group used ∼98% of the glucose. However, it was observed that metformin caused more glucose consumption not only at the end of the third day but also every day of measurement (Fig. 4).

### Effect of metformin on intracellular ATP concentration

**Figure 5.**
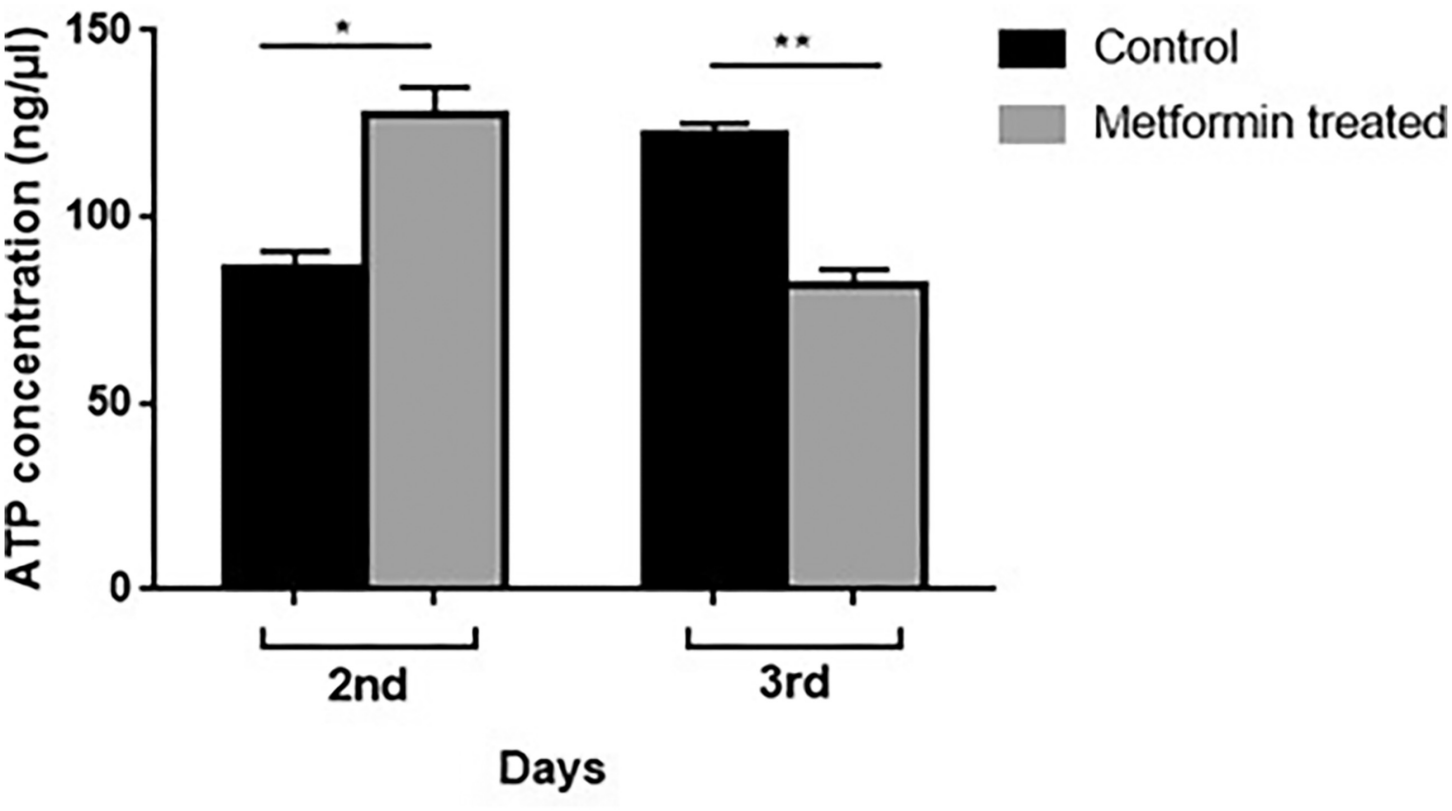
Metformin increased the ATP production in cells grown in a 3% SD medium. A total of 10^6^ cells were collected at 48th and 72nd hours, then centrifuged, washed twice with sterile distilled water, and disrupted in 100 μl ATP assay buffer using a dismembrator. ATP concentrations were determined using an ATP colorimetric assay kit (Sigma-Aldrich/MAK190) and the measurements were performed according to the manufacturer’s instructions. Error bars represent the ranges of duplicate experiments. *P < .05, **P < .01.

Based on the finding that metformin increases glucose consumption, intracellular ATP concentration was measured on the second and third day of cultures to see if metformin causes a change in ATP production. It was observed that 25 mM metformin increased the ATP concentration by ∼30% on the second day, while it decreased by ∼26% on the third day (Fig. 5).

### Measurement of intracellular oxidation levels

To determine the effect of metformin on the amount of ROS that increases with aging, cell cultures grown in liquid media without metformin (control group) and with metformin were taken as samples on the second and third days, and the levels of intracellular total oxidation and lipid peroxidation were measured.

**Figure 6.**
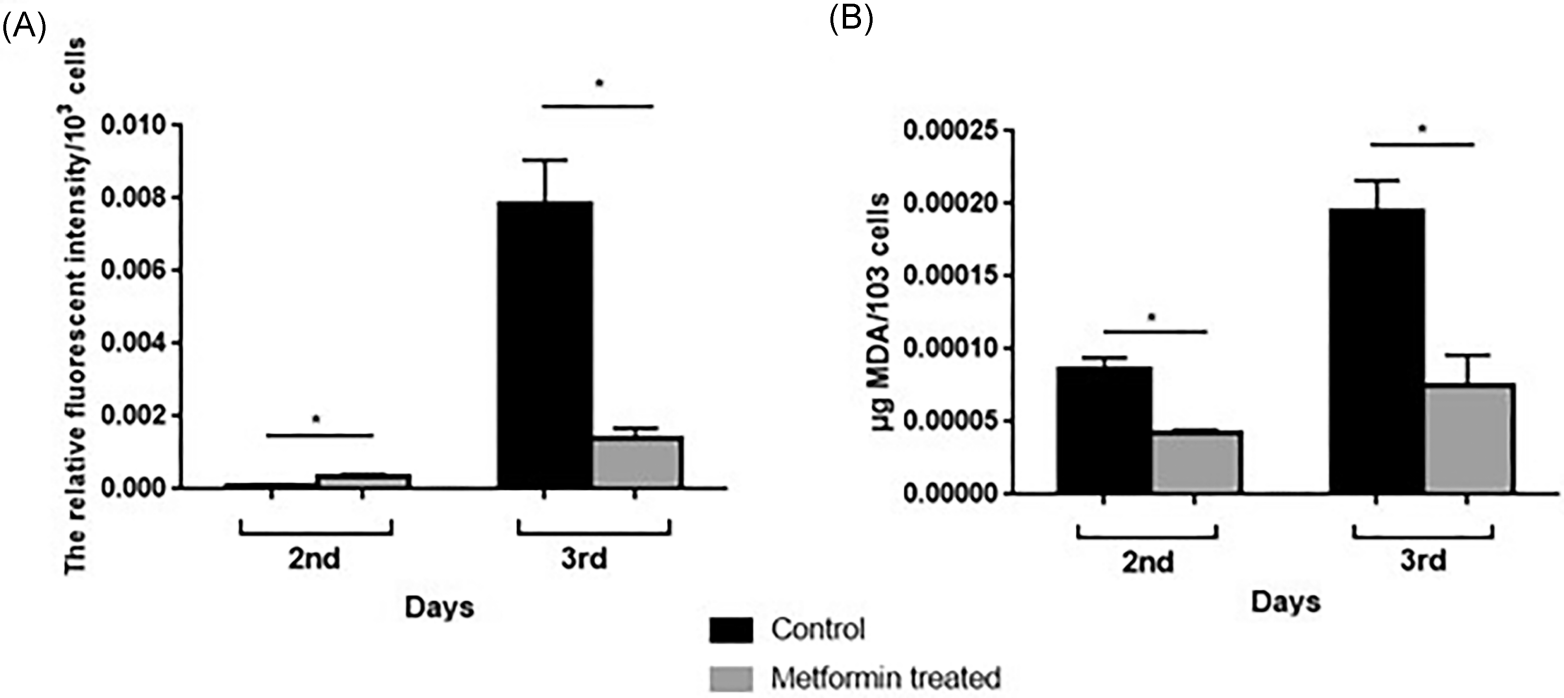
Metformin reduced total intracellular oxidation (A) and lipid peroxidation (B) levels. Measurements were done using the samples taken from the same culture and as described in the “Materials and Methods” section. Error bars represent the ranges of duplicate experiments. *P < .05.

In terms of total intracellular oxidation, only a slight difference was observed between the control and metformin groups on the second day, while metformin reduced ROS formation in the cells by ∼74% on the third day compared to the control group (Fig. 6A). When we assess the concentration of MDA, the end-product of the lipid peroxidation, it was observed that metformin reduced lipid peroxidation in cells by 50% on the second day and ∼38% on the third day compared to the control group (Fig. 6B).

### Comparison of protein carbonylation levels

**Figure 7.**
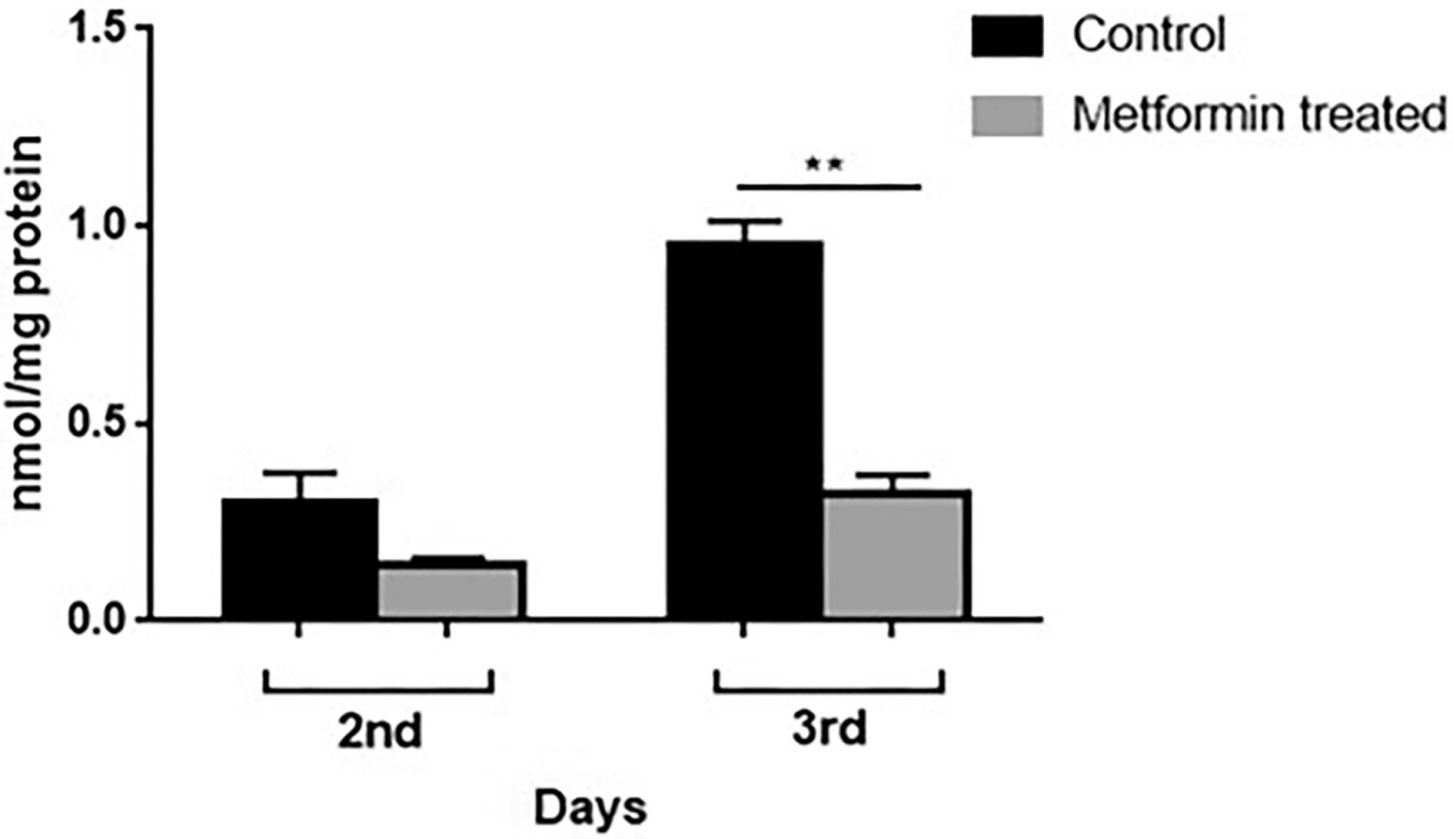
Metformin prevented the increase of protein carbon level on the third day. Measurements were done as described in the “Materials and Methods.” There was no difference between the control and metformin groups on the second day of inoculation. However, on the third day, the protein carbonyl levels of the control group increased by ∼60%, while the metformin carbonyl level remained unchanged. **P < .01.

Based on the fact that the loss of protein homeostasis is one of the hallmarks of aging, we wanted to assess the protein carbonylation levels, which can be informative on the state of homeostasis. When the data of the second and third days of culture were compared, no significant difference was observed between the control and metformin groups on the second day, while the amount of carbonylated protein in the cells treated with 25 mM metformin on the third day was ∼35% less than the control group (Fig. 7).

### Evaluation of the protective role of metformin against hydrogen peroxide and heat stress

**Figure 8.**
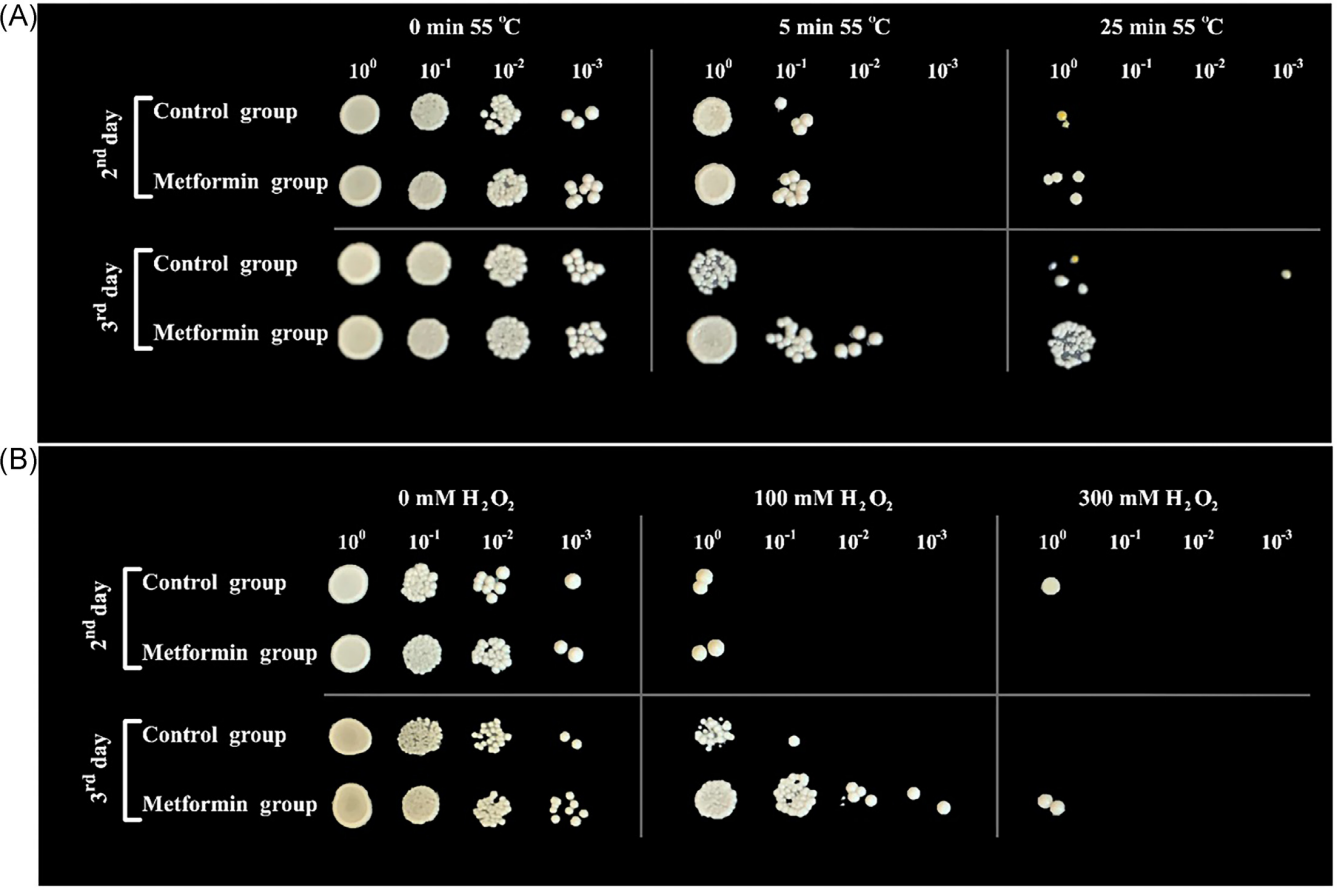
Metformin-treated long-lived cells showed increased stress resistance. (A) Resistance to heat stress. Samples from metformin-treated and untreated cells were taken on the second and third day of inoculation, washed with sterile water, and 5 × 10^6^ cells were resuspended in sterilized Milli-Q water then heat shocked at 55°C for different lengths of time. Then placed on ice for 2 min, 10-fold serial dilutions were made, and 5 μl aliquots of each suspension were spotted onto the rich medium. (B) Resistance to H_2_O_2_. Cells from the same two groups as in (A) were resuspended in varying concentrations of H_2_O_2_ and incubated at 30°C for 90 min then diluted and spotted on plates.

It is known that long-lived cells (i.e. cells that are subjected to calorie restriction) in various organisms can cope with the environmental stress more successfully than those grown under standard conditions (Sinclair 2005). To determine if the metformin-treated, long-lived cells have increased resistance to environmental stress, we tested the viability of cells that were exposed to H_2_O_2_ (100 and 300 mM for 90 min) and heat stress (55°C for 5 and 25 min). We observed that metformin protected cells against H_2_O_2_ and heat stress in all the tested conditions (Fig. 8).

## Discussion

### Metformin increases the lifespan of *S. pombe* depending on the day of addition and is also effective in glucose-free medium

The fact that metformin acts on all nine of the hallmarks of aging increases the number of studies to fully understand its antiaging potential (Kulkarni et al. 2020). In this study, we aimed to expand the findings on the effects of metformin at the cellular and molecular level by using *S. pombe*, which has emerged as an important model organism in understanding the aging process.

First, we found that 25 mM metformin increased lifespan by ∼50% compared to untreated cells. Onken and Driscoll (2010) reported that 50 mM metformin increases the mean lifespan of *C. elegans* by 27%. In another study (Anisimov et al. 2005), metformin (100 mg/kg) added to the drinking water of female transgenic HER-2/neu mice increased the mean lifespan by 8% and the maximum lifespan by 1 month compared to the control group; in SHR mice, on the other hand, treatment of females with the same dose of metformin resulted in a 37% prolongation of the mean lifespan and a 2.8-month prolongation of the maximum lifespan compared to the control group. Numerous studies have been conducted on the lifespan prolonging effect of metformin given at different doses and different stages of the life of various organisms and obtained different results. For example, Anisimov et al. (2011) showed that metformin effects on lifespan in mice are age-dependent. Accordingly, we added metformin to media on different days to see if the same was true for *S. pombe*. Metformin added to the culture from the beginning extended the lifespan as mentioned, but when added especially on the third day of the culture it had no favorable effects on the cells. It has been reported that the positive effect of metformin on CLS requires the presence of extracellular glucose (Borklu-Yucel et al. 2015). Therefore, it can be inferred that the ineffectiveness of lately added metformin on *S. pombe* is due to the depletion of almost all the glucose in the medium on the third day (Fig. 4) and the corresponding decrease in ATP production (Fig. 5). While metformin had favorable effects in younger individuals of *C. elegans*, it shortened the lifespan of older ones (Espada et al. 2020) and it was suggested that the toxicity was linked to lower levels of ATP and aggravation of aging associated mitochondrial dysfunction toward respiratory failure in these older individuals. Besides, interventions that stabilize ATP levels alleviate late life metformin toxicity. On the other hand, it should be kept in mind that *S. pombe* is not a multicellular organism, the effects of metformin are evaluated only at the cellular level, and factors other than cellular mechanisms play a role in the effectiveness of metformin in multicellular organisms.

To see if there can be another mechanism for the lifespan prolonging effect of metformin other than that depending on glucose availability, we grew cells in a glucose-free medium containing 25 mM metformin. Although the cells live longer in the glucose-free medium (fasting condition) than in the medium containing 3% glucose, these nondividing cells survived much longer in the glucose-free medium containing metformin. It was reported that under fasting, *S. pombe* cells had significantly decreased antioxidant capacity and they lost energy compounds, such as glutathione and ATP (Pluskal et al. 2011). In addition, mutation levels in quiescent *S. pombe* cells increased where DNA lesions are repaired with errors (Gangloff et al. 2017). Although underlying mechanisms are not clear regarding how it maintains genome stability and regulates genotoxic oxidative damage, it was shown that metformin reduced micronuclei and chromosomal aberrations, activated DNA base excision repair system, and recruited DNA repair complexes at the DNA double-strand breaks in various cell types (Vazquez-Martin et al. 2011, Dogan Turacli et al. 2018, Cheki et al. 2021). Thus, the lifespan-extending effect of metformin in the fasting condition may have resulted from its stimulation of DNA damage responses and oxidative-stress prevention mechanisms, which are not dependent on glucose availability. On the other hand, it should be noted that the medium we used in this study was not the minimal medium used in the studies with *S. pombe* mentioned above, and the cells in the quiescent state are caused by the absence of glucose, not nitrogen. Considering the similarity of *S. pombe* to mammalian cells in terms of cellular features such as cell cycle control, cell division, and DNA repair and recombination (Hoffman et al. 2015), it will be important to investigate the mechanisms by which metformin affects these processes in *S. pombe*.

### Metformin increases glucose consumption and ATP production

To examine the glucose consumption in metformin-treated cells, we measured the remaining glucose in the medium on first, second, and third days of growth. We observed that metformin caused more glucose consumption than the control group on the first (2-fold), second (25,7%), and third days (9,2%) of inoculation. In other words, while the cells in the control group consumed 85.6% of the glucose in the medium at the end of the third day, the metformin-treated group consumed 93.5% of the glucose. It was observed in rodents that metformin increased glucose uptake into the cell by 218% by suppressing SHIP2 protein, whose expression is increased in diabetic rodent models, and which causes an increase in insulin resistance and a decrease in glucose uptake as a result of suppressing insulin signaling (Polianskyte-Prause et al. 2019). Metformin is known to increase glucose uptake into cells by promoting the translocation of GLUT family glucose transporter receptors (Fischer et al. 1995, Yang and Holman 2006). In a recent study, phloretin, a broad-spectrum GLUT transport inhibitor, reduced glucose uptake in metformin-treated cells. This reveals that GLUT transporters are responsible for glucose uptake and that metformin also acts through these transporters in glucose uptake into the cell (Yang et al. 2021). Metformin was shown to upregulate genes encoding glucose transporters in *S. cerevisiae* (Borklu-Yucel et al. 2015). Further studies are needed to evaluate the role of glucose transporters and related mechanisms in metformin-treated cells in *S. pombe*.

A common effect shared by calorie restriction and metformin is the activation of the AMPK pathway, which plays a central role in the regulation of energy metabolism. Although it is not clear how metformin activates AMPK, it decreases ATP production by suppressing complex I in the mitochondrial electron transport chain (El-Mir et al. 2000, Owen et al. 2000) and increases the amount of AMP by inhibiting AMP deaminase (Ouyang et al. 2011) suggesting that the drug may indirectly activate AMPK. In this study, we observed that the metformin group produced more ATP on the second day than the control group, but on the third day, ATP levels were lower than in the control group. Foretz et al. (2010) observed that metformin reduces mitochondrial ATP synthesis in hepatocytes. Buler et al. (2012) treated mouse primary hepatocytes with different doses of metformin for up to 72 hours and demonstrated that ATP levels decreased as a result of metformin administration, in agreement with previous data. However, Yang and Holman (2006) showed that metformin increased the glycolytic capacity of intestinal cells and increased ATP production due to glycolysis and lactate release. Moreover, studies in humans showed that metformin can activate mitochondrial respiration (Victor et al. 2015). In line with this, pharmacological metformin concentration increased the ATP levels in primary hepatocytes (Wang et al. 2019). These suggest that the contradictory results are related to the applied metformin dose. Fermentation is promoted and aerobic respiration is suppressed in yeast cells (Crabtree effect) growing in nutrient-rich environments (Alexander and Jeffries 1990). However, the metformin dose that we used may have increased aerobic respiratory capacity on the second day, similar to the above-mentioned pharmacological dose. Besides, the fact that *S. pombe* is less sensitive to glucose suppression (Zuin et al. 2008) may be a facilitating factor in increasing aerobic respiratory capacity by metformin. On the third day, the amount of ATP produced decreased compared to the control group possibly due to the depletion of almost all the glucose in the medium.

### Metformin-treated cells have decreased levels of free radicals and show increased stress resistance

As mentioned above, many studies have shown metformin’s genome protective effects via lowering ROS or by indirect scavenging mechanism (Fontaine 2018). It is thought that metformin exerts its antioxidative effect by suppressing mitochondrial complex I, also known as type I NADH dehydrogenase (Batandier et al. 2006), and increasing antioxidant gene expression via the SKN-1/Nrf2 transcription pathway (Onken and Driscoll 2010). Metformin’s reduction of ROS level is not only mediated by inhibition of the mitochondrial respiratory chain, but also by suppression of nicotinamide adenine dinucleotide phosphate (NADPH) oxidase (Zainabadi 2018). Metformin has been shown to reduce ROS production through inhibition of NADPH oxidase activity in colorectal cancer cells (Nguyen et al. 2019). We observed that metformin decreased the amount of total intracellular ROS production (Fig. 6A) and the amount of lipid peroxidation (Fig. 6B) and carbonylated protein (Fig. 7), especially on the third day in *S. pombe*. Metformin has been shown to significantly reduce age-induced ROS, lipid peroxidation, carbonylated protein, and acetylcholinesterase in rat erythrocytes and liver cells (Sadeghi et al. 2019). Mitochondrial genes for mitochondrial complex I are absent in *S. pombe*; instead, there are two nuclear genes encoding NADH dehydrogenases that function as mitochondrial complex I (Chiron et al. 2007). This suggests that there may be two alternative ways for metformin to reduce ROS production and related oxidative damage in *S. pombe*. Metformin may reduce oxidative damage by acting either through NADH dehydrogenases that function as mitochondrial complex I or through a mechanism other than mitochondrial complex I. Note that, although *S. pombe* grows mainly by fermentation in the presence of glucose, it is much more susceptible than budding yeast to mutations and mitochondrial damage that affect mitochondrial functions (Malecki et al. 2016). Therefore, the beneficial effects of metformin on oxidative damage may be of greater importance in increasing CLS in *S. pombe*, among other effects.

The ability to mount an effective response to environmental and cellular stress factors plays an important role in determining the aging process and the onset and progression of aging-related diseases. For example, studies have shown that long-lived mutant strains in nematodes and fruit flies are often more resistant to more than one type of stress (Larsen 1993, Lithgow et al. 1995, Lin et al. 1998, Cheng et al. 2003, de Castro et al. 2004). Chen and Runge (2009) compared the stress resistance of cells grown on media containing 3% and 0.1% glucose and showed that the stress resistance of calorie-restricted *S. pombe* cells was higher than that of cells grown under normal conditions. We observed that metformin protected cells against both oxidative and heat stress, especially on the third day suggesting that metformin behaved as a caloric restriction mimetic.

This study offers preliminary insights that contribute to the understanding of metformin’s antiaging properties in *S. pombe*. Taken together, our results suggest metformin exerts its lifespan-extending effects in *S. pombe* partly through energy regulation and stress resistance mechanisms that were also observed in various organisms. Results also imply that *S. pombe* can be used as an effective model organism to shed more light on the antiaging properties of metformin. To gain a more detailed understanding of these mechanisms, further studies can be made using mutant *S. pombe* cells in terms of genes involved in the basic pathways affected by metformin, and the effects of the drug on the aging process can be elucidated from a broader perspective with genomic studies such as transcriptome, proteome, and epigenetics analysis.
